# Modelling reading development through phonological decoding and self-teaching: implications for dyslexia

**DOI:** 10.1098/rstb.2012.0397

**Published:** 2014-01-19

**Authors:** Johannes C. Ziegler, Conrad Perry, Marco Zorzi

**Affiliations:** 1 Laboratoire de Psychologie Cognitive, Aix-Marseille University and Centre National de la Recherche Scientifique, Fédération de Recherche 3C, Brain and Language Research Institute, 3 place Victor Hugo, 13331 Marseille, France; 2 Faculty of Life and Social Sciences, Swinburne University of Technology, John Street, Hawthorn, Victoria 3122, Australia; 3 Dipartimento di Psicologia Generale, Università di Padova, Via Venezia, 8-35131 Padova, Italy; 4 IRCCS San Camillo Neurorehabilitation Hospital, Via Alberoni 70, 30126 Venice, Italy

**Keywords:** phonological decoding, developmental dyslexia, computational modelling, reading development

## Abstract

The most influential theory of learning to read is based on the idea that children rely on phonological decoding skills to learn novel words. According to the self-teaching hypothesis, each successful decoding encounter with an unfamiliar word provides an opportunity to acquire word-specific orthographic information that is the foundation of skilled word recognition. Therefore, phonological decoding acts as a self-teaching mechanism or ‘built-in teacher’. However, all previous connectionist models have learned the task of reading aloud through exposure to a very large corpus of spelling–sound pairs, where an ‘external’ teacher supplies the pronunciation of all words that should be learnt. Such a supervised training regimen is highly implausible. Here, we implement and test the developmentally plausible phonological decoding self-teaching hypothesis in the context of the connectionist dual process model. In a series of simulations, we provide a proof of concept that this mechanism works. The model was able to acquire word-specific orthographic representations for more than 25 000 words even though it started with only a small number of grapheme–phoneme correspondences. We then show how visual and phoneme deficits that are present at the outset of reading development can cause dyslexia in the course of reading development.

## 1. Introduction

Reading development is fundamentally a process in which novel orthographic codes have to be mapped onto pre-existing phonological codes (spoken words), which are associated to meaning prior to reading [1]. The initial stages of this process are characterized by learning how letters and groups of letters map onto their corresponding sounds. This process is referred to as *phonological decoding* and allows children to recode words that they have heard but never seen before, thus giving them access to the thousands of words that are present in their spoken lexicons [2]. In theory, every successfully decoded word provides the child with an opportunity to set up direct connections between a given letter string (orthography) and the spoken word [2,3], which results in the development of an orthographic lexicon. Phonological decoding thus provides a powerful self-teaching device because the explicit learning of a small set of spelling–sound correspondences allows the child to decode an increasingly large number of words, which bootstraps orthographic and lexical development [2,4,5]. We refer to this learning loop as the *phonological decoding self-teaching* (PDST) hypothesis.

No existing computational model of reading has tried to capture this fundamental learning loop (see below). Thus, how decoding based on an initially small number of spelling–sound correspondences, for example grapheme–phoneme relationships, would allow the system to correctly retrieve whole word phonology and set up connections between letter strings and entries in an orthographic lexicon (orthographic development) has not been explored. Importantly, as pointed out by Share [2], this learning loop operates in a self-teaching fashion. That is, no external teacher provides correct teaching signals for thousands of words but the child simply decodes based on a small set of spelling-to-sound correspondences, and it is the decoded word itself which provides the teaching signal to the model. In this respect, it is particularly important to investigate what happens when words are decoded incorrectly. Is self-teaching possible with a non-optimal initial decoding process? How is reading development affected by deficits that are present during these initial stages of reading development? Given that dyslexia is a development disorder, simulations of the precise learning mechanisms are crucial in furthering our understanding of it. This article tries to tackle these issues.

A number of previous models have been proposed to capture reading development and to simulate dyslexia [6–9], but none of them have tried to implement the developmentally plausible PDST hypothesis described above. The most influential learning model was based on the parallel distributed processing approach [8,10]. Harm & Seidenberg [6] set up a three layer network that learnt to map orthography onto a pretrained phonological attractor network representing the child's initial knowledge about phonological structure. The model was trained by providing the orthography of about 3000 words and then propagating the discrepancy (error) between the predicted and the actual phonology back to the weights between the orthographic, hidden and phonological layers. Although the model was able to learn 99% of the training set after 10 million trials, it is obvious that this ‘massive’ learning process is very different from a developmentally plausible theory of reading development. Most importantly, in order to learn, the model requires an ‘external teacher’, which provides correct teaching signals on millions of learning trials. By contrast, the PDST hypothesis suggests that the explicit teaching of a small number of spelling-to-sound mappings is at the start of reading development. These initially rudimentary decoding skills, in combination with phonological representations of spoken words available prior to reading, provide the system with an *internally* generated teaching signal, which gradually improves decoding and bootstraps orthographic and lexical development.

A somewhat different approach to modelling reading aloud has been proposed by Perry, Zorzi and Ziegler in the context of the connectionist dual processing (CDP) model [7,11–13]. This model has two processes, a non-lexical one that maps orthography to phonology in a two-layer associative (TLA) network, and a lexical one that connects orthography to phonology in a hard-wired interactive activation network. The non-lexical TLA network learns *linear* relationships between strings of graphemes and strings of phonemes very quickly [14]. Therefore, it can read nonwords but may produce the incorrect phonology for words with spelling–sound relationships that are either ambiguous or difficult to decode. By contrast, the direct and hard-wired interactive activation network links the orthographic entries of words to their phonological counterparts. Therefore, it can read any type of word, but not nonwords. In normal conditions, output from the two processes is integrated to jointly determine reading aloud. With regard to the objectives outlined above, it is important to note that Perry *et al*. [7,11] have not yet explored whether basic phonological decoding via the TLA network can bootstrap orthographic and lexical development, especially under conditions in which the correct output is not provided through an external teaching signal (i.e. self-teaching). In other words, the question remains open as to whether phonological decoding initially based on a small number of grapheme–phoneme correspondences can activate correct word candidates in the phonological lexicon and whether self-teaching in the absence of externally provided teaching signals is sufficient to support stable learning and orthographic development.

This study has two parts. In the first part, we implement and test the PDST hypothesis in the context of the CDP model. In the second part, we explore how deficits in this learning loop would give rise to the reading impairments seen in dyslexic children. Ultimately, this research will allow us to make simulations of reading outcomes for individual children or groups of children on the basis of their underlying deficits with a developmentally plausible model.

## 2. Computational investigation of reading development

**Figure 1.**
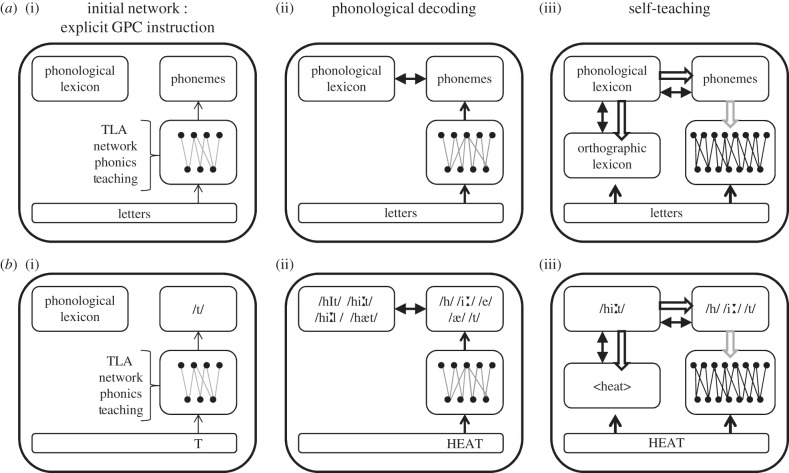
(*a*,*b*) Illustration of the phonological decoding and self-teaching mechanisms in the context of the CDP [13] model. After initial explicit teaching on a small set of grapheme–phoneme correspondences (GPCs), for example T->/t/ (i), the network is able to decode novel words, for example HEAT (ii), which has a pre-existing representation in the phonological lexicon. If the decoding mechanism activates a word in the phonological lexicon (here, the correct word /hi:t/ is more active than its competitors), an orthographic entry is created (<heat>) and the phonology of the ‘winner’ (/hi:t/) is used as an internally generated teaching signal (grey arrows) to improve and strengthen the weights of the TLA network (iii).

The basic architecture of the model and the PDST learning loop are presented in figure 1. Given that children know a large number of spoken words prior to reading, we assume that the phonological lexicon is in place before training starts (initial network). Consistent with the idea that the initial steps of reading are characterized by the explicit teaching of basic spelling–sound correspondences, the TLA network was pretrained on a small set of grapheme–phoneme correspondences similar to those found in common phonics programmes, for example *Jolly Phonics* (for details, see [15]). Next, we presented the TLA network with written words to be learnt. On the basis of the pretraining, the TLA network computed the potential (but possibly incorrect) pronunciation of a novel word, which typically results in the activation of word units in the phonological lexicon through feedback from the phonemes to the phonological lexicon. If a word entry is found in the phonological lexicon which is consistent with the letter string, a direct connection is set up between the written word and its phonological counterpart (orthographic development). That is, the word becomes *lexicalized*. In turn, the internally activated phonology of the word is then used as a training signal to adjust the weights of the TLA network (i.e. self-teaching). The TLA network is trained with the delta rule, which is formally equivalent to the Rescorla–Wagner learning rule, which has been widely used to account for human learning [16,17]. Importantly, the use of the delta rule makes learning of the spelling–sound mappings in the TLA network extremely quick. This means that there is already a lot of learning happening in a few hundred learning trials [14], as opposed to the millions of trials needed to train a multi-layer backpropagation model (i.e. [6]). Thus, every successful decoding event has two consequences: (i) it is used to set up direct connections between the letter string and the whole word phonology, and (ii) it improves the decoding mechanisms of the TLA network. This learning loop is illustrated in figure 1 (see figure legend for a detailed description).

### (a) Simulation 1

In this simulation, we tested the basic PDST mechanism described above with the exception that we assumed that a child can choose the correct phonology among the cohort of activated units in the phonological lexicon through context, semantics or syntactical constraints. This is, of course, an oversimplification but not an unrealistic one because during the initial stages of learning-to-decode children have a lot of information which helps them to select the correct word, such as images in story books, short sentences with constraining context, paired reading and feedback from the teacher. After pretraining, the TLA network was presented with 32 735 words (all of the words used in [12]). We considered a word had been learned correctly if the correct phonological entry was found in the cohort of activated neighbours, in which case its corresponding orthographic representation was set up in the orthographic lexicon. Thus, each learning trial can establish a representation in the orthographic lexicon. The dynamics of the lexical route are identical to those implemented in previous CDP+ models (i.e. interactive activation), and to simplify things, each time a connection was set up, the resting threshold of the word node, which is designed to represent the frequency at which the word occurs, was set to its log frequency in the same way as it is in the CDP+ models. Note that the word node threshold could be replaced by a self-feedback connection that is strengthened at each word encounter [18], thereby providing a dynamic and learning-based account of the frequency effect without major changes to the model's lexical route

**Figure 2.**
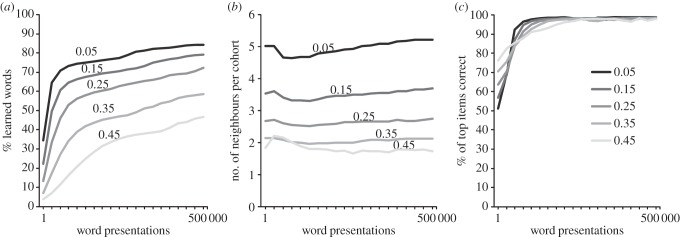
Simulations of learning to read through phonological decoding and self-teaching. Performance of the network using five different word recognition thresholds (0.05, 0.15, 0.25, 0.35 and 0.45). (*a*) Percentage of learnt/lexicalized words; (*b*) numbers of neighbours in the cohort of each recognized word and (*c*) percentage of items in each cohort where the correct item was also the most strongly activated item.

In order to facilitate the activation of word units in the phonological lexicon, we reduced the phoneme-phonology inhibition parameter (to −0.02) so that items in the phonological lexicon were easier to activate than in the skilled reading model [7]. To investigate the performance of the model in a parametric way, we chose five word recognition thresholds at which a word in the phonological lexicon was considered activated enough to be recognized (0.05, 0.15, 0.25, 0.35 and 0.45). All models were run for 500 000 word presentations.^1^ The results are shown in figure 2.

As can be seen from figure 2, with low word recognition thresholds (i.e. where words in the phonological lexicon need less activation to become activated), the model learnt most of the words despite the fact that it started off with only a small set of grapheme–phoneme relationships learnt during pretraining. For instance, with a word recognition threshold of 0.05, the model successfully learnt more than 80% of the words. This percentage is actually very high given the large number of words with ambiguous spelling–sound correspondences, which cannot be decoded correctly using the linear TLA network [7,15]. Figure 2*b* shows the numbers of co-activated neighbours in the cohort of each recognized word. The results show that the number of neighbours activated above the criterion was relatively small—vastly less than the cohort of all possible neighbours. Figure 2*c* shows the proportion of items in each cohort where the correct item was the most active. As can be seen, very rapidly in the course of learning, the most active item tends to be the correct word, which is the reason why self-teaching can work so well. In summary, this simulation provides a *proof of concept* for the claim that phonological decoding and self-teaching provide a powerful bootstrapping mechanism [2] which allows the beginning reader to ‘start small’ (i.e. with a small set of explicitly taught letter–sound correspondences) and to build upon this knowledge to ‘self-learn’ the majority of words (up to 80%) through a simple decoding mechanism that gets more efficient with every successfully decoded word.

### (b) Simulation 2

**Figure 3.**
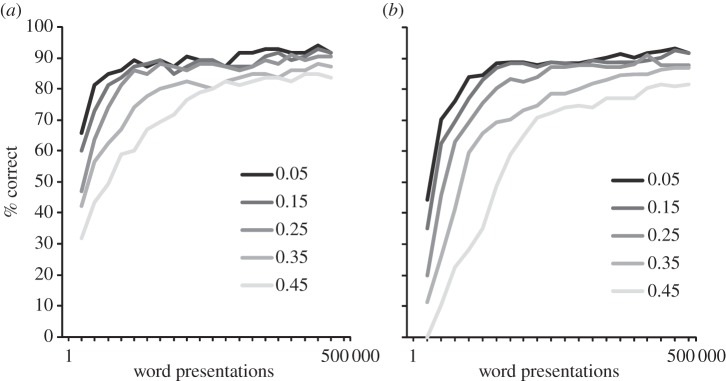
Can the model read novel words? Generalization performance of the model on the nonwords of Olson *et al*. [19] and Rastle & Coltheart [20]. The nonword sets were repeatedly tested during the course of learning to read (i.e. after every 50 000 word presentations).

When evaluating a learning model, the most important question is always whether such a model can *generalize* its learned knowledge to novel items. In the case of reading, generalization is simply tested by presenting the model with nonwords that the model has never seen before. Nonword reading performance was assessed by presenting the model with the nonwords of Olson *et al*. [19] and Rastle & Coltheart [20]. The first set was chosen because it has been extensively used to investigate performance of children with and without dyslexia [21]. The second set was used because it represents an exceptionally hard set of nonwords [7,11]. To study the developmental trajectory of nonword generalization, the list of nonwords was presented to the model after every 25 000 word presentations during the course of learning to read. Nonword pronunciations were considered correct if the output of the TLA network (i.e. phoneme buffer) corresponded to any grapheme–phoneme or body–rime relationship that exists in real words. The results of these simulations are shown in figure 3. As can be seen, the model quickly yields very good generalization performance, which supports the conclusion that the implemented PDST learning loop is sufficient to decode novel words with high accuracy.

### (c) Simulation 3

One important question is what would happen if an incorrect word were lexicalized. In other words, if phonological decoding results in the activation of an incorrect word, to what extent would such imperfections perturb the rest of the learning process (i.e. does it cause catastrophic interference?). This is the hardest and most realistic test of the PDST hypothesis because it is reasonable to assume that a child will sometimes fail to select the correct word among the activated word candidates in a given cohort (figure 2*b*).

**Figure 4.**
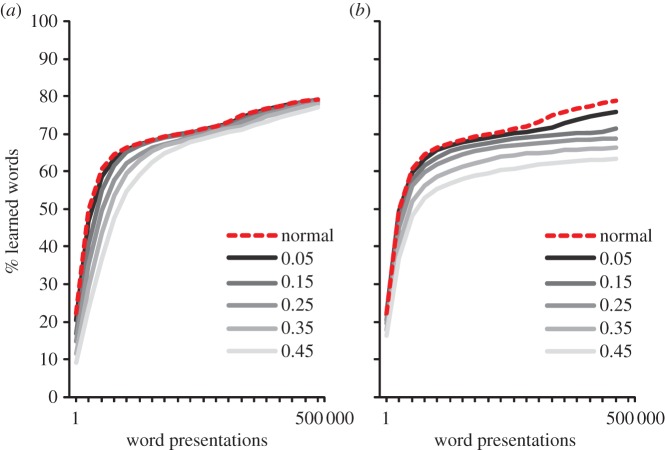
What happens when decoding goes off track? Network performance when the model fails to learn a given word (no learning, (*a*)) or when an incorrect item has been learnt (incorrect learning, (*b*)). This happened with a probability of 0.05, 0.15, 0.25, 0.35 and 0.45. The dotted line represents the unimpaired network. All simulations are run with a word recognition threshold of 0.15. See the text for details. (Online version in colour.)

This was explored in two conditions: ‘No Learning’ and ‘Incorrect Learning’. In the no-learning condition, it was assumed that children do not have enough semantic, syntactic or contextual information available to choose the target word from the cohort of activated words. To simulate this, instead of adding a correctly decoded word to the orthographic lexicon and then training the TLA network on it, nothing was done with the word (i.e. no learning occurred). The probability of this happening was manipulated parametrically with a probability of 0.05, 0.15, 0.25, 0.35 and 0.45. In the incorrect-learning condition, we went one step further and assumed that an *incorrect* word was lexicalized and learned. That is, when a word was found in the phonological but not in the orthographic lexicon, rather than train the model on the correct word and then lexicalize it, we randomly chose any word from the activated cohort and trained the TLA network on it. Again, this was manipulated parametrically with a probability of 0.05, 0.15, 0.25, 0.35 and 0.45. All simulations were run with a word recognition threshold of 0.15. The results are shown in figure 4.

As can be seen in figure 4*a*, the manipulation where a certain percentage of items were not lexicalized (the no-learning condition) did not appear to affect the results much. Even when almost half the words were missed (0.45 probability), it seems that learning was simply slowed down, with accuracy reaching the same asymptote as the unimpaired model. In the incorrect-learning condition (figure 4*b*), while there was an overall drop in performance caused by training the model on the incorrect pronunciations, even when this was done almost half the time (0.45), the model was still able to correctly learn more than half of words in the database.

The results from the no-learning condition are not so surprising because if a word is not found once, it may be found on the next attempt. This suggests that it is important for children to read words in different contexts—if one context fails, another may work. This supports the idea that contextual diversity plays an important role in reading beyond word frequency [22]. The reasonable performance of the model even when trained on incorrect words (incorrect-learning condition) shows that the model is very error tolerant, and thus can cope with the type of decoding errors children might make (e.g. choosing *beer* for *bear*). Together then, both simulations suggest that failing to choose a word correctly and even choosing words incorrectly are not serious problems for the PDST model. This strongly supports the developmental plausibility of this kind of model.

## 3. Computational investigation of developmental dyslexia

Having implemented a developmentally plausible and functioning learning loop, we can now ask how different deficits might affect the learning-to-read process. The literature on developmental dyslexia highlights at least two core deficits, which can be identified prior to reading. The first is related to phonological processing deficits that are most apparent in phonological awareness tasks [23,24]. This deficit seems to be universal as it is found across transparent and opaque writing systems [25,26]. The second deficit is related to visual and orthographic processing difficulties that can be seen in tasks where children have to process letter strings that are not pronounceable, for example RWTXN [27–30]. Recent evidence suggests that such letter-in-string processing deficits might result from abnormally strong crowding [31] or poor visual-attentional processing [32], which might be identified even prior to reading [33].

In the following simulations, we take the unique opportunity to investigate how deficits that are present prior to learning to read affect the learning-to-read process itself. This allows us to look at the *causal* link between a specific deficit and the reading outcome across development akin to a longitudinal study. Clearly, the advantage of a simulation study compared to a longitudinal study with children is that we can manipulate the nature and the severity of single underlying deficits. Below, the effects of visual and phonological deficits are simulated both on word learning (Simulation 4) and generalization performance (Simulation 5).

### (a) Simulation 4

To simulate visual difficulties, each letter in a word was switched with the letter next to it with a certain probability (0.02, 0.04, 0.06, 0.08 and 0.10). Thus, for example, instead of presenting CAT to the model, we would present ACT. Such letter position errors are relatively frequent in children with dyslexia [28,34].

**Figure 5.**
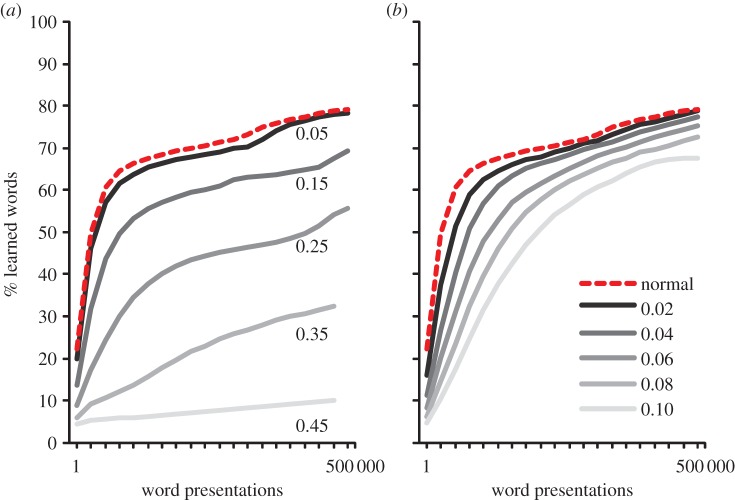
Learning to read with phoneme or visual deficits. (*a*) Phoneme deficits were simulated by changing a correctly assembled phoneme with a phonetically similar but incorrect phoneme with a certain probability (0.05, 0.15, 0.25, 0.35 and 0.45). (*b*) Visual deficits were simulated by switching a letter with the letter next to it with a certain probability (0.02, 0.04, 0.06, 0.08 and 0.10). The dotted line represents the unimpaired network. All simulations were run with a word recognition threshold of 0.15. (Online version in colour.)

To simulate deficits in phonological awareness, each time a correct word was activated in the phonological lexicon, we changed the phonemes in the output of the TLA network, which resulted in an incorrect teaching signal. Again, this was done parametrically by changing each correct phoneme with a certain probability (0.05, 0.15, 0.25, 0.35 and 0.45). Changing phonemes was not done randomly, but rather, the correct phoneme was turned off and another was turned on as a function of how many distinctive features were shared between the two (e.g. /b/ was more often switched to /p/ than to /s/, because /b/ and /p/ only differ on voicing)^2^, although we never chose phonemes with more than three different features. The results are presented in figure 5. As can be seen, the effect of the two deficits on performance varied in a non-additive way across the levels of impairments. Basically, the greater the deficit, the more it deteriorated the learning performance of the model. That was especially so for the phonological deficits, where the model with the strongest deficit had very low performance. The deteriorated performance of the phoneme-deficit model contrasts in an intriguing way with the relatively spared performance of the incorrect-learning simulation^3^ (Simulation 3, figure 4*b*). The most obvious reason for the difference is that when an incorrect word is selected from a cohort, it typically has overlap with the correct phonology. Thus, even if many words are swapped, most of the phonology the model is trained on is still correct. Alternatively, with the phonological impairment, the phonemes are changed to something entirely different, which results in very poor performance. The visual deficits also affect the learning process. When comparing the two simulations, it would be tempting to conclude that visual deficits have a somewhat smaller impact than phoneme deficits. However, in the absence of real data, which would allow us to estimate the size of the underlying deficit for each child [35], such a conclusion would be premature.

### (b) Simulation 5

Impaired reading in developmental dyslexia is particularly clear when children have to read nonwords [36]. Again, nonword reading deficits are present both in opaque and transparent writing systems [37]. Poor nonword reading suggests an inefficient decoding mechanism, which prevents stable orthographic learning as outlined above. To investigate the effects of visual and phoneme deficits on nonwords reading during the course of reading development, we examined generalization performance on the same set of nonwords and in the same way as in Simulation 2. The severity of the two types of impairments was manipulated parametrically as in Simulation 4.

**Figure 6.**
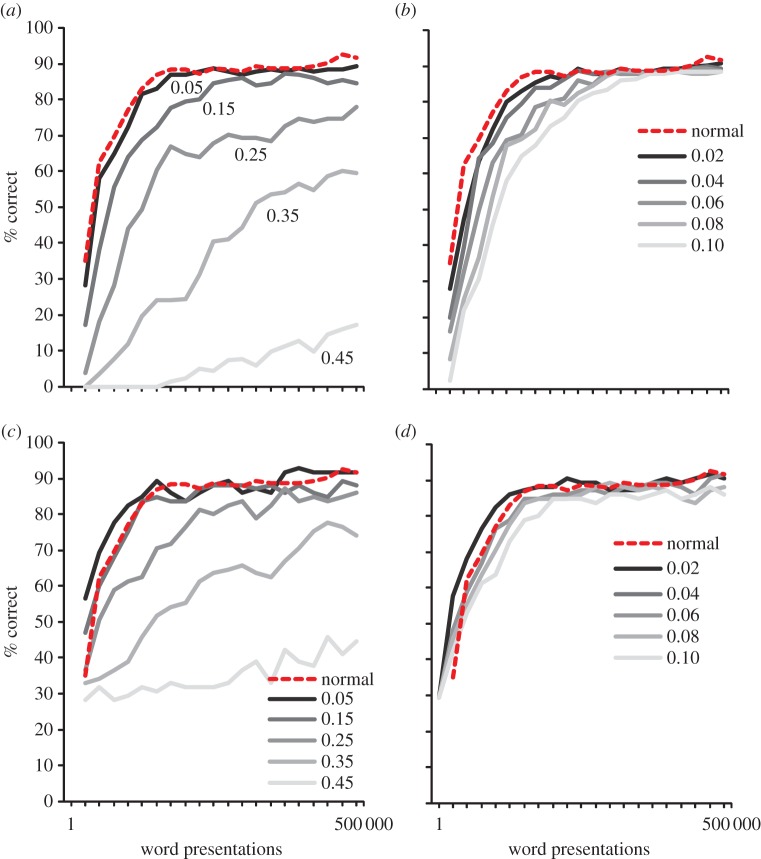
(*a*,*c*) Effects of phoneme and (*b*,*d*) visual deficits on nonword reading. (*a*,*b*) Simulations with the relatively hard nonwords of Rastle & Coltheart [20]. (*c*,*d*) Simulations for the relatively easy nonwords of Olson *et al*. [19]. The dotted line represents the unimpaired network. All simulations were run with a word recognition threshold of 0.15. (Online version in colour.)

The results are presented in figure 6. As can be seen, the results showed that phoneme deficits had a strongly negative effect on generalization performance on the easy [19] as well as the hard set of nonwords [20]. With the present levels of impairments, the visual deficits had a much weaker effect on generalization performance. Again, this might be a function of the level of impairment that was chosen.

## 4. Discussion

The most influential theory of learning to read is based on the idea that children rely on basic phonological decoding skills to learn words they have heard but never seen before [2]. According to Share's [2] self-teaching hypothesis, ‘each successful decoding encounter with an unfamiliar word provides an opportunity to acquire the word-specific orthographic information that is the foundation of skilled word recognition’ (p. 155). A relatively small number of successful exposures appear to be sufficient for acquiring orthographic word representations [5]. Therefore, phonological decoding acts as a self-teaching mechanism or ‘built-in teacher’ [2]—this is thought to be the principal means by which the learner attains word recognition proficiency in all alphabetic writing systems [1,38].

The major contribution of the present article is a proof of concept that the implementation of the PDST hypothesis works in the context of a real computational model of learning to read. As we have shown in the simulations, such a model is able to acquire word-specific orthographic representations for more than 25 000 words and read aloud novel words even when starting with a relatively small number of grapheme–phoneme correspondences. Thus, this work provides the first developmentally plausible computational model of reading development.

Indeed, all previous connectionist models of reading [6–8,11,39] have learned the task of reading aloud through the exposure to a very large corpus of spelling–sound pairs. That is, the input (spelling) and the ‘desired’ output (target pronunciation) for many thousands of words are typically presented until the error-correction procedure employed as learning algorithm reaches a level of performance that is considered adequate by some external criterion. However, this training regimen is highly implausible: the kind of supervised learning used in all models implies that a teacher externally supplies the pronunciation of all words that should be learnt. As argued above, in real life, although there is an external teacher (sometimes), the external teacher does not provide correct pronunciations for many thousands of words. As a matter of fact, the power of self-teaching is the idea that such an external teacher is not needed [2].

In this work, an external teacher is only needed for the pretraining of a small number of grapheme–phoneme correspondences [15] and for the selection of some word candidates during the initial stages of learning. This process reflects real classroom teaching, which necessarily starts with the explicit (supervised) instruction of a small set of grapheme–phoneme correspondences (i.e. phonics). From there on, the model ‘is left alone’. That is, on the basis of these rudimentary decoding skills, the model will produce pronunciations for unfamiliar words. If a word is found in the phonological lexicon but is not yet in the orthographic lexicon, a direct link between the two is established. Thus, exactly as in Share's [2] theory, each successful decoding encounter with an unfamiliar word provides an opportunity to acquire word-specific orthographic information. At the same time, the pronunciation of the decoded word is used as a ‘built-in-teacher’—that is, an internally generated teaching signal—to improve the efficiency of the TLA decoding network itself. As a result, high decoding accuracy is obtained rather quickly (figure 3).

One important issue that we have not fully addressed yet is what happens when initial decoding results in the activation of several word candidates. In our simulations, we simply chose the correct word (figure 2*c*) if it was in the cohort of word candidates. This oversimplification is based on the assumption that in the real learning situation with real texts, children will have additional information from the story context, images, semantics or syntax to help them chose the correct target. Nevertheless, as shown in Simulation 3, even if the model failed to choose a word or chose an incorrect word, the learning process was not dramatically affected (figure 4), because such errors might be rectified on subsequent encounters of the same word. This suggests that it is important for a child to read words in different contexts.

One important concern is how words that do not get activated via a phonological loop will ever get into the lexicon. This might be a somewhat ‘anglocentric’ problem [40] because of the relatively large number of words with inconsistent or ambiguous spelling-to-sound correspondences. Clearly, it would be much less of a problem in transparent writing systems, for example Italian, where phonological decoding based on a few grapheme–phoneme correspondences activates unique word candidates with high accuracy [15]. Despite the relatively high level of inconsistency, it is worth noting that the phonological decoding network was still able to learn up to 80% of the words. The remaining 20% have and typically will be learnt through different strategies, for example rote learning [4]. Fortunately enough, many irregular words are very frequent (*dead*, *have*, *done*, *come*…) and, therefore, can be easily taught in an explicit and supervised fashion during primary school. A second issue is how words that are not in the phonological lexicon will ever get there. This is not a fundamental problem because one can assume that, once the decoding mechanism has become efficient, every phonologically decoded word will create an entry in the phonological lexicon (if it is not already there), which will be strengthened with every additional encounter of the same word (i.e. vocabulary acquisition through reading).

The upshot of having a fully implemented developmental model of learning to read is that such a model can be used to investigate how deficits that are present prior to reading or occur during reading development might cause the kind of reading impairments seen in children with dyslexia (e.g. slow reading, poor decoding, letter confusion errors, etc.). In Simulations 4 and 5, we have shown that the model can potentially explain how two of the most established deficits—visual and phoneme deficits—affect orthographic development and nonword reading. In future work, we will attempt to use real data [21], which allows us to estimate the size of the underlying deficit(s) for each individual child and then investigate to what extent impairments that mimic those of dyslexics would predict inter-individual differences and dyslexia subtypes (see [35] for a similar approach using a model of skilled reading that does not learn).

It will be of major interest to contrast the effects of various kinds of deficits. For example, phonological deficits can be implemented through poor vocabulary (a small phonological lexicon), noise in the phonological lexicon, underspecified phonological representations or phoneme deficits. Similarly, visual deficits could be simulated through noisy letter detectors, poor letter position coding or crowding effects that would affect some letter positions more than others. Note that it is important to also investigate the combination of deficits, which are unlikely to be additive [35]. Interestingly, some genetic analyses suggest that a single factor, best described as a genetically determined learning-rate factor, underlies decoding, spelling and orthographic learning [41]. In our model, *learning rate* is one of the key parameters, which can be modified individually to explore how inter-individual differences in learning rate might affect decoding and orthographic learning. Along the same lines, *noisy computation* could be a common factor, which might affect the quality of representations and the efficiency of the learning process. This could be implemented by adding a certain amount of noise non-specifically at all levels of the model.

If this work is successful, the model could be used to predict developmental trajectories for at-risk children before dyslexia is actually diagnosed [42]. It could also be used to develop and assess (through simulations) optimal sequences and materials for reading and intervention programmes. In sum, the implementation of a developmentally plausible learning model might not only help us to understand the heterogeneity of dyslexia (i.e. how various kinds of impairments and their interactions give rise to different dyslexia phenotypes) but might fundamentally change the way we go about models of skilled reading [43].

## Endnotes

1 Training the networks on 500 000 events is not implausible. Harry Potter's *Order of the Phoenix* contains about 257 000 words. Thus, a child who reads all seven Harry Potter volumes exceeds by far the number of training trials used in our simulations.

2 We used 23 distinctive features to describe the phonemes. Fifteen of these were for consonants: syllabic, consonantal, sonorant, continuant, delayed release, sibilant, voiced, nasal, high, back, anterior, labial, coronal, distributed and lateral. Eight were for vowels: high, low, front, back, round, tense, offglide and onglide.

3 This is a fair comparison because the initial grapheme–phoneme pretraining was identical (unimpaired) in both cases.
